# Independent Predictors of 28-Day Mortality and the Critical Role of Source Control in *Stenotrophomonas maltophilia* Bacteremia in the ICU

**DOI:** 10.3390/pathogens15040364

**Published:** 2026-03-30

**Authors:** Mustafa Uğuz, Berfin Çirkin Doruk

**Affiliations:** Department of Infectious Diseases and Clinical Microbiology, Mersin City Training and Research Hospital, 33240 Mersin, Türkiye; berfin.cirkindoruk@saglik.gov.tr

**Keywords:** *S. maltophilia*, bloodstream infection, intensive care unit, antimicrobial resistance, mortality predictors, prognostic biomarkers

## Abstract

*Stenotrophomonas maltophilia* bacteremia is an opportunistic infection associated with high mortality among intensive care unit (ICU) patients, largely due to intrinsic antimicrobial resistance and the severe underlying conditions of affected hosts. This study aimed to identify independent predictors of 28-day mortality, evaluate the prognostic value of laboratory biomarkers, and assess the impact of source control in ICU patients with *S. maltophilia* bacteremia. A retrospective single-center case–control study was conducted over a five-year period, including 148 ICU patients with *S. maltophilia* bacteremia and 1:1 matched non-*S. maltophilia* controls. Demographic, clinical, and laboratory data were analyzed using multivariate logistic regression to determine independent predictors of mortality, while receiver operating characteristic (ROC) analysis assessed the prognostic performance of selected biomarkers. Multivariate analysis identified shock (OR = 6.01; 95% CI: 3.13–11.56; *p* < 0.001), underlying malignancy (OR = 4.31; 95% CI: 1.44–12.96; *p* = 0.009), higher Sequential Organ Failure Assessment (SOFA) score (OR = 1.28; 95% CI: 1.16–1.41; *p* < 0.001), and elevated procalcitonin levels (OR = 1.014; 95% CI: 1.002–1.026; *p* = 0.019) as independent predictors of mortality. A multivariable prediction model incorporating age, SOFA score, shock, malignancy, procalcitonin, and lactate demonstrated good discriminative performance for predicting 28-day mortality (AUC = 0.817; *p* < 0.001). Source control was significantly associated with improved survival. These findings suggest that hemodynamic instability, malignancy, and severe organ dysfunction are major determinants of mortality in ICU patients with *S. maltophilia* bacteremia, and that multidimensional risk assessment models integrating clinical severity scores and biomarkers may facilitate early identification of high-risk patients and support targeted clinical management.

## 1. Introduction

Bacteremia refers to the presence of bacteria in the bloodstream, which may be transient and not always associated with clinical symptoms, whereas septicemia implies a systemic inflammatory response with organ dysfunction. In ICU settings, bloodstream infections remain a major cause of morbidity and mortality [1]. In the intensive care setting, the widespread use of invasive devices, broad-spectrum antibiotics, and the high severity of patients’ illnesses create an environment conducive to the emergence of challenging hospital-acquired pathogens. The increasing rates of antimicrobial resistance are making treatment more complex, leading clinicians to turn to different therapeutic agents. However, the current pace of developing effective agents does not seem to be keeping up with the rate at which options are diminishing [2].

*Stenotrophomonas maltophilia* (*S. maltophilia*) is a Gram-negative, non-fermenting bacterium commonly found in human habitats, including water and soil. Despite being widely known as a low-virulence colonizer, it has become an important opportunistic pathogen [3]. It is characterized by its remarkable resilience, allowing it to colonize diverse hospital reservoirs. Especially in critically ill and immunocompromised patients, it is increasingly recognized as an opportunistic pathogen associated with nosocomial infections, particularly ventilator-associated pneumonia and catheter-related bloodstream infections [4].

*S. maltophilia* possesses two inducible L1 and L2 beta-lactamase enzymes and an active efflux pump system, granting it intrinsic resistance to numerous critical antibiotic classes, most notably carbapenems [5]. It also possesses a high capacity for biofilm production on indwelling medical devices, which physically shields the bacteria from antibiotics and host immune defenses [6]. Such special abilities contribute to mortality rates for *S. maltophilia* bacteremia, which range between 20% and 70% across international cohorts [7,8,9].

Although the high mortality rate of *S. maltophilia* bacteremia has been documented in numerous studies, there is still no consensus on a definitive, evidence-based guideline that includes risk classification for clinical practice. The current literature generally consists of single-center studies and presents the results of analyses that involve different patient populations [10]. When considered individually, these studies have critical gaps in terms of definitive source control and prognostic tools, particularly with regard to the inclusion of modifiable factors. It is essential to develop a valid guideline that defines the clinical impact by including specific comorbidities, such as solid malignancy, as key variables. In addition to this, the guideline should quantitatively demonstrate the critical impact of the timing and success of source control strategies, particularly catheter removal. It should also determine the threshold values of clinical and laboratory biomarkers. The implementation of such a comprehensive guideline might have the potential to improve patient outcomes and optimize early treatment decisions as a targeted preventive tool in the treatment of patients with *S. maltophilia* bacteremia.

## 2. Methods

### 2.1. Design and Patients

This retrospective, single-center case–control study was conducted at Mersin City Training and Research Hospital, a large tertiary-care referral center located in Mersin, a major city in the Mediterranean region of Türkiye. The hospital has approximately 2200 beds and includes multiple specialized intensive care units, providing advanced care for critically ill patients and serving as a referral center for surrounding provinces. This study included patients admitted between 1 January 2020, and 31 December 2024.

Patients in the study group were identified through the hospital electronic database as ICU patients with *S. maltophilia* bacteremia. Adult patients (≥18 years) with at least two blood cultures positive for *S. maltophilia* were included. Patients with only a single positive blood culture, polymicrobial bacteremia, transfer/discharge/death within 48 h after the index culture, or incomplete clinical data were excluded. The index date was defined as the date of the first blood culture positive for *S. maltophilia* (day 0). Controls were selected from ICU patients hospitalized during the same period without *S. maltophilia* growth in blood cultures.

Patient files and the hospital information system were reviewed to record demographic characteristics, comorbidities, history of immunosuppressive therapy, hospital admission in the last six months, antibiotic use in the last six months, invasive procedures (central venous catheter, intubation, tracheostomy, drain, and percutaneous enteral gastrostomy), and concomitant infections. Clinical outcomes included time to initiation of appropriate antimicrobial therapy, duration to negative blood culture, and in-hospital and 28-day mortality rates. Patients with missing data for more than 5% of the variables of interest were excluded from the analysis.

For each patient, the data extracted from the electronic medical records included age, gender, history of noncommunicable chronic diseases (chronic obstructive pulmonary, cardiac, renal diseases, etc.), history and presence of malignancy, Sequential Organ Failure Assessment (SOFA) score, Acute Physiology and Chronic Health Evaluation II (APACHE II) score, Charlson Comorbidity Index (CCI) score, episodes of hypotension and shock, and microbiology and biochemistry laboratory test results. Clinical and laboratory parameters were assessed twice, at the time of the first positive culture growth for *S. maltophilia* and seven days later. The seventh day was selected for assessment in order to demonstrate the clinical response and short-term changes in laboratory markers during the early phase of antimicrobial treatment [11].

The primary outcome was 28-day all-cause mortality, which was defined as death from any cause within 28 days following the date of the culture result with first positive growth for *S. maltophilia.*

### 2.2. Definitions

Catheter-related bloodstream infection (CLABSI) was defined according to CDC/NHSN criteria [12]. Laboratory-confirmed bloodstream infection was defined as the growth of *S. maltophilia* in at least two separate blood cultures consistent with clinical findings. Mortality was defined as death from any cause during the follow-up period. SOFA and APACHE II scores were used to assess disease severity. The Sequential Organ Failure Assessment (SOFA) score evaluates the extent of organ dysfunction across multiple systems, whereas the Acute Physiology and Chronic Health Evaluation II (APACHE II) score is a widely used severity-of-disease classification system that incorporates physiological parameters, age, and chronic health conditions to estimate the risk of mortality in critically ill patients.

### 2.3. Microbiological Characterization

Isolates were identified using the VITEK^®^ 2 Compact system (bioMérieux, Marcy l’Étoile, France). Antimicrobial susceptibility testing was performed and interpreted according to the Clinical and Laboratory Standards Institute (CLSI) criteria. Susceptibility to trimethoprim–sulfamethoxazole and levofloxacin was specifically evaluated.

### 2.4. Statistics

Initially, in order to control for inherent selection bias and confounding by indication—specifically, based on the observation that *S. maltophilia* infections frequently occur in patients with higher baseline disease severity—a propensity score matching (PSM) analysis was performed. Propensity scores were estimated using a multivariable logistic regression model. The model included covariates known to influence both the likelihood of infection and the mortality: age, gender, APACHE II score, SOFA score, presence of malignancy, presence of dialysis requirement, and baseline shock status. Cases were matched using a 1:1 “nearest neighbor” algorithm without replacement. The quality of the match was assessed using Standardized Mean Differences (SMDs), where an SMD < 0.1 was considered indicative of negligible imbalance between the groups. The PSM was performed using the RStudio 2 September 2025 “Cucumberleaf Sunflower” Release for Microsoft Windows, and the following statistical analyses were performed using SPSS Statistics v27 (IBM, Armonk, NY, USA). Continuous variables were expressed as the mean ± standard deviation (SD) or median (interquartile range) and compared using the Student’s *t*-test or Mann–Whitney U test. Categorical variables were compared using the Chi-square or Fisher’s exact test. Variables found to be significant in the univariate analysis or deemed clinically relevant (e.g., shock presence) were entered into a binary logistic regression model to identify independent risk factors for 28-day mortality. Results were reported as odds ratios (ORs) with 95% confidence intervals (CIs). The impact of catheter removal on survival was analyzed using Kaplan–Meier survival curves, with differences assessed by the log-rank test. The predictive power of key laboratory values (e.g., lactate and SOFA) for 28-day mortality was assessed using Receiver Operating Characteristic (ROC) curve analysis, with the area under the curve (AUC) reported. A *p*-value of <0.05 was considered statistically significant for all tests.

## 3. Results

**Figure 1:**
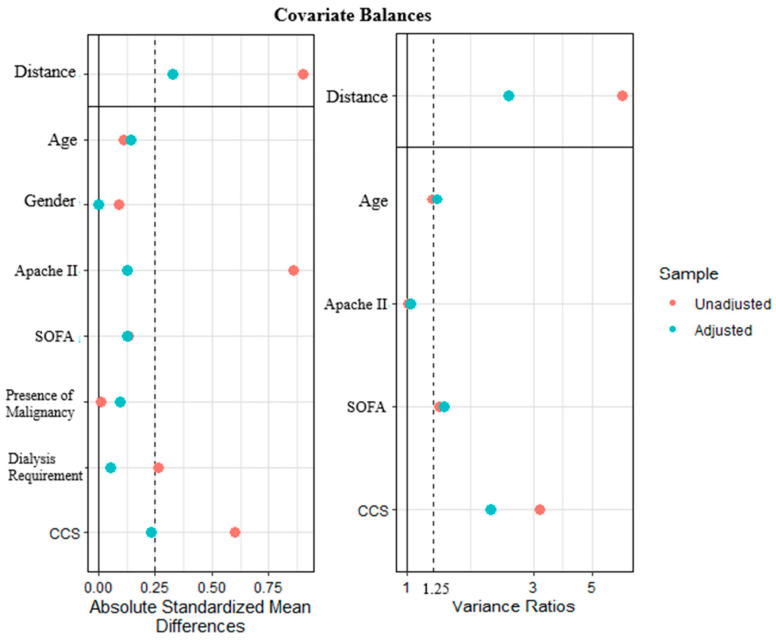
Standardized mean difference and variance ratio plots for the cohort before and after propensity score matching.

Initially, there were a total of 148 study cases and 1036 control cases. After PSM, a total of 296 subjects were included in the statistical analysis, consisting of 148 patients with *S. maltophilia* and 148 severity-matched controls. The standardized mean difference (SMD) for age, gender, APACHE II, and presence of malignancy was less than 0.1, while those for critical severity markers, including the SOFA score and shock status, were below 0.25, which was considered an acceptable threshold (Figure 1).

Prior to analysis, the normality of all continuous data was assessed using the Kolmogorov–Smirnov and Shapiro–Wilk tests. All continuous clinical and laboratory variables demonstrated non-normal distributions (*p* < 0.05). Consequently, non-parametric statistical methods were applied. Among the 296 patients analyzed, the population had a mean age of 66.7 years (IQR: 18, range: 20–99). Clinical severity at the time of admission was high, as evidenced by a median APACHE II score of 12 (IQR: 12) and a median SOFA score of 7 (IQR: 4). The patients were monitored for a median of 6 culture-negative days (IQR: 12). Regarding invasive interventions, the median duration of catheterization was 10 days (IQR: 14). The median procalcitonin levels decreased from 22.0 ng/mL (IQR: 35.7) at baseline (PCT1) to 0.53 ng/mL (IQR: 2.44) at the second measurement (PCT2). Similarly, the median CRP levels showed a reduction from 15.9 mg/L (IQR: 13.8) to 4.0 mg/L (IQR: 7.1). Nutritional and metabolic markers remained critical; the median albumin levels were 2.63 g/dL (IQR: 0.90) at baseline and 2.90 g/dL (IQR: 0.76) at follow-up. The median phosphorus levels were 11.0 mg/dL (IQR: 12.0) at the time of the first sampling (Table S1).

No statistically significant differences were observed between the study and control groups in terms of age, APACHE II, SOFA, and CCI scores, as well as duration of dialysis (*p* > 0.05). Regarding clinical course variables, the duration of catheterization was significantly shorter in the study group compared to the control group (*p* < 0.001), whereas the number of culture-negative days was significantly longer in the study group (*p* < 0.001) (Table 1).

The assessment of the initial laboratory measurements (Day 1) revealed that serum phosphorus (*p* < 0.001) and procalcitonin (*p* < 0.001) levels were significantly higher in the study group compared to the control group. There were no statistical differences observed between the two groups in other baseline laboratory values (albumin, total protein, ALT, AST, CRP, calcium, creatinine, lymphocyte, neutrophil, white blood cell count (WBC), erythrocyte sedimentation rate (ESR), and lactate) (*p* > 0.05).

In the follow-up laboratory measurements (Day 7), differences in certain clinical markers were more evident. AST (*p* = 0.047), CRP (*p* = 0.041), phosphorus (*p* < 0.001), procalcitonin (*p* < 0.001), and lactate (*p* = 0.028) values in the study group were found to be significantly higher when compared to the control group. There were no significant differences between the groups regarding the second measurements of albumin, total protein, ALT, calcium, creatinine, lymphocytes, neutrophils, WBC, and ESR (*p* > 0.05). The comparative analysis results are presented in Table 2.

The relationship between the groups (control vs. study) and the clinical outcomes (survival vs. mortality) was evaluated using a Chi-square test. In the control group, 50% (*n* = 74) of the patients survived, while 50% (*n* = 74) resulted in mortality. Similarly, in the study group, 52.7% (*n* = 78) of the patients survived, and 47.3% (*n* = 70) resulted in mortality. Statistical analysis revealed no significant association between the treatment type and patient outcome (*x*^2^ = 0.216, df = 1, *p* = 0.642). The categorical variables compared between the study and control groups are presented in Table 3.

The Mann-Whitney U test, based on the outcomes, indicated that age and SOFA score differed significantly (Table 4A). Also, creatinine, lactate, and procalcitonin levels differed significantly (Table 4B).

A Spearman’s rank–order correlation analysis was conducted to evaluate the relationships between significantly associated clinical parameters. The analysis revealed a significant positive correlation between the outcome and several disease severity indicators, notably the SOFA score (r_s_ = 0.350, *p* < 0.001) and shock status (r_s_ = 0.334, *p* < 0.001). Furthermore, the outcome was significantly positively correlated with baseline lactate (r_s_ = 0.195, *p* = 0.001), follow-up lactate (r_s_ = 0.123, *p* = 0.034), presence of malignancy (r_s_ = 0.194, *p* = 0.001), and age (r_s_ = 0.135, *p* = 0.020). Significant relationships were also observed among the clinical course variables: the number of culture-negative days showed a strong positive correlation with initial phosphorus levels (r_s_ = 0.554, *p* < 0.001) and a negative correlation with shock status (r_s_ = −0.211, *p* < 0.001). Additionally, catheter duration was positively correlated with shock status (r_s_ = 0.375, *p* < 0.001), while it exhibited significant negative correlations with both culture-negative days (r_s_ = −0.315, *p* < 0.001) and initial phosphorus levels (r_s_ = −0.334, *p* < 0.001) (Table 5).

A series of multivariable logistic regression models was developed. The final multivariable logistic regression model identified four independent predictors of mortality. After adjusting for confounders, the presence of shock (Exp(B) = 6.014, *p* < 0.001) and the presence of malignancy (Exp(B) = 4.314, *p* = 0.009) were the strongest predictors. Additionally, higher SOFA scores (Exp(B) = 1.276, *p* < 0.001) and initial procalcitonin levels (Exp(B) = 1.014, *p* = 0.019), phosphorus levels (Exp(B) = 1.013, *p* = 0.073), and lactate levels (Exp(B) = 1.139, *p* = 0.085) showed a trend toward an association with mortality (Table 6).

**Figure 2:**
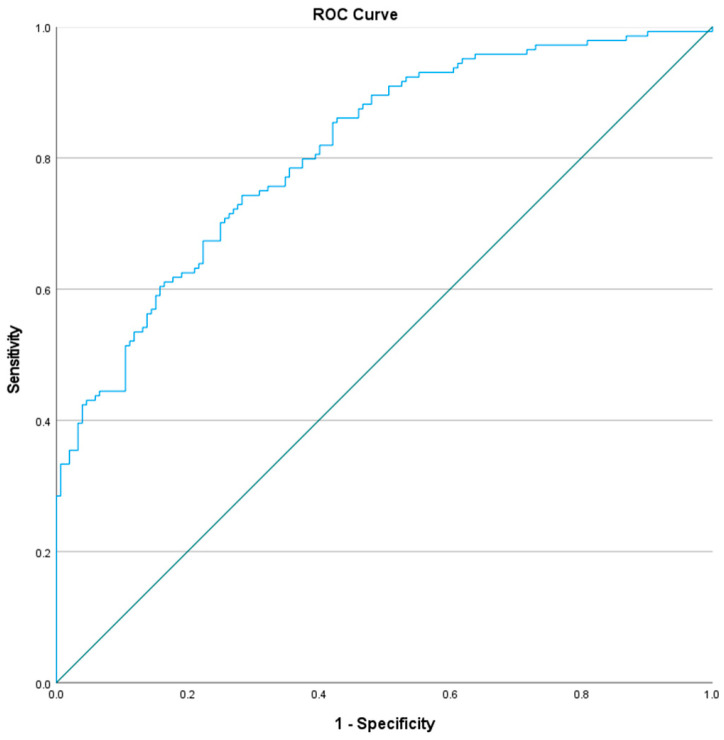
The ROC curve showed an AUC of 0.817 and an overall model quality of 0.76, using the predicted probabilities involving SOFA, shock, malignancy, procalcitonin, age, and lactate.

ROC curve analysis was performed to determine the overall prognostic accuracy of the multivariable logistic regression model, which was performed using the predicted probabilities derived from the final model (incorporating age, SOFA, shock status, malignancy, procalcitonin, and lactate). The model demonstrated discriminative power, with an AUC of 0.817 and an overall model quality of 0.76 (Figure 2).

## 4. Discussion

The objective of this retrospective case-control study was to evaluate the clinical characteristics and identify independent predictors of mortality in ICU patients with *Stenotrophomonas maltophilia* bacteremia. Propensity score matching was used to reduce the confounding effects of baseline disease severity, enabling a sound comparison between *S. maltophilia* patients and matched control cases. Our findings indicate that shock, underlying malignancy, and high SOFA scores are the strongest independent predictors of mortality.

Our findings indicate that hemodynamic instability, which is characterized by shock, emerged as the most significant predictor of fatal outcomes, with a six-fold increase in mortality risk. This highlights the critical importance of early recognition and resuscitation in ICU patients who develop *S. maltophilia* infections. Consequently, the SOFA score was found to be significantly higher in non-survivors. The analysis results showed that the SOFA score was an independent predictor of mortality (OR = 1.276; 95% CI: 1.155–1.409; *p* < 0.001). The strong predictive capacity observed in this study is consistent with the existing literature, which indicates that elevated SOFA scores are associated with an increased risk of ICU mortality, irrespective of the underlying pathogen [13]. In the presence of *S. maltophilia* bacteremia, the baseline organ reserve is often already compromised. Therefore, an elevated SOFA score at the onset of bacteremia indicates severe systemic impairment and a weak host response, which can result in a fatal outcome. These findings emphasize that early and continuous longitudinal assessment of organ dysfunction using the SOFA score is crucial for risk stratification and the prompt initiation of aggressive, targeted therapies in this high-risk patient population.

The presence of malignancy was associated with an over four-fold increase in the risk of death. *S. maltophilia* is a recognized opportunistic pathogen that disproportionately affects immunocompromised hosts, particularly those with solid organ malignancies. The immunosuppressive nature of both the underlying disease and antineoplastic therapies likely impairs the host’s ability to clear the infection, leading to worse clinical outcomes. In a study conducted by Zhang et al., the authors demonstrated that high levels of *S. maltophilia* were positively associated with cancer progression and a poor prognosis. Specifically, patients in the *S. maltophilia*-high group exhibited higher rates of tumor recurrence and death. Furthermore, the two-year survival rate was significantly lower in patients with elevated *S. maltophilia* levels, underscoring its potential role as a prognostic biomarker for adverse clinical outcomes and pathogenesis in patients with underlying malignancies [14].

Analysis of laboratory parameters revealed that non-survivors had significantly higher baseline lactate levels (median: 3.3, IQR: 2.0 vs. 2.8, and IQR: 1.48; *p* = 0.001) and procalcitonin levels (median: 26, IQR: 49.3 vs. 19, and IQR: 37.9; *p* = 0.036) compared to survivors, but no significant difference was observed in baseline creatinine levels between non-survivors and survivors (median: 1.48, IQR: 2.8 vs. 1.63, IQR: 2.19; *p* = 0.944). Elevated initial lactate, indicative of tissue hypoperfusion and altered metabolism, correlated significantly with mortality. However, while serum phosphorus and procalcitonin were markedly elevated in the *S. maltophilia* group compared to the controls, their independent predictive value for mortality diminished when adjusting for profound variables like shock and the SOFA score in the multivariate model.

Regarding the clinical course, patients with *S. maltophilia* bacteremia had a longer time to culture negativity but a significantly shorter catheterization duration compared to the control group (median: 10 days, IQR: 12 vs. 11 days, IQR: 7; *p* < 0.001). In the context of duration of catheterization, the strong, time-dependent relationship between mortality rates and permanent devices is an important mechanistic insight focused on biofilm formation. Given that *S. maltophilia* is a notorious biofilm producer that readily adheres to plastic surfaces, the resulting extracellular polymeric substance matrix physically impedes antibiotic penetration and creates a unique microenvironment that alters bacterial physiology, rendering even sensitive antibiotics ineffective. Such protection from both antibiotics and the host immune system (phagocytic and complement attack) makes in situ eradication extremely difficult [15]. Since biofilm protects the pathogen, definitive source control—removal of the colonized foreign body—is the most effective way to eliminate the high bacterial load and allow systemic antibiotics to eradicate the remaining circulating bacteria [16]. Our findings are consistent with the recommendations, which indicate that the threshold for removing a CVC in a patient with *S. maltophilia* bacteremia should be exceptionally low.

In our analysis, initial serum phosphorus levels demonstrated a trend toward significance in predicting mortality (OR = 1.013; 95% CI: 0.999–1.028; *p* = 0.073) but did not emerge as an independent prognostic factor in the multivariate model. In the context of critical illness and severe sepsis, elevated serum phosphorus (hyperphosphatemia) is predominantly driven by acute kidney injury, which drastically impairs renal phosphate excretion. Additionally, widespread tissue ischemia and cell lysis secondary to shock release abundant intracellular phosphate stores into the systemic circulation [17]. Although initial comparisons indicated significantly elevated levels of phosphorus, its independent predictive value was outweighed by more comprehensive markers of systemic collapse, such as the SOFA score and the presence of shock. The SOFA score is designed to evaluate renal dysfunction, as indicated by creatinine and urine output, which plays an important role in phosphate metabolism [18]. Consequently, the observation of hyperphosphatemia is suggestive of its role as a marker for the severity of renal impairment and cellular hypoxia, rather than an independent contributor to mortality. Nevertheless, Al Harbi et al. have highlighted a significant relation between phosphorus levels and ICU mortality among septic shock cases [19]. Concurrently, a recent systematic review and meta-analysis report, including 38,320 cases, stated that a high serum phosphate level was associated with an elevated all-cause mortality risk (RR = 1.46; 95% CI [1.22–1.74]; *p* > 0.001) [20].

The independent predictive strength of markers like procalcitonin (*p* = 0.019, OR = 1.014), alongside shock and SOFA, emphasizes a reliance on systemic inflammatory profiles over individual electrolyte fluctuations in outcome forecasting. In parallel, Sezen et al. recently examined a cohort of 87 *S. maltophilia* cases and indicated that procalcitonin levels were significantly higher in deceased patients, suggesting that initial alterations in procalcitonin should be considered as effective markers of refractory bacterial sepsis [21].

Several limitations of this study should be acknowledged. The retrospective design inherently limits the ability to establish strict causal relationships. Also, due to the rigorous data collection phase, the initial and early follow-up biochemical and clinical parameters included in this study cover a limited number of markers. Other biological markers that play an important role in the pathophysiology and prognosis of infection, such as cytokines, inflammatory mediators, or bacterial virulence factors, were not included in this study. The absence of data related to the antimicrobial treatment regimens and treatment initiation times may be considered another limitation. On the other hand, they may show heterogeneity among practices and might be considered as a potential factor that could affect clinical outcomes. The single-center nature of this study limits its ability to generalize the findings to other institutions with distinct local microbiologic ecologies or resistance patterns. Moreover, it is important to point out the limitations inherent in the propensity score matching applied to the cohort initially obtained at the beginning of this study. Although this study was conducted at a single center, the findings should be interpreted with caution because the retrospective design and the possibility of residual confounding may limit their generalizability. Finally, due to the focus on *S. maltophilia* bacteremia, a rare pathogen, the sample size may have been limited for some subgroup analyses. In order to establish more definitive findings and strengthen the evidence base for clinical decision-making, larger, multicenter prospective studies are necessary.

### Strengths and Limitations

This study has several strengths. First, the use of propensity score matching minimized baseline differences between the study and control groups, allowing a more balanced comparison and reducing potential confounding related to disease severity. Second, the comprehensive evaluation of clinical severity scores, laboratory biomarkers, and source control strategies provided a multidimensional assessment of factors associated with mortality in ICU patients with *Stenotrophomonas maltophilia* bacteremia. Third, the inclusion of dynamic laboratory measurements during follow-up allowed evaluation of early clinical changes in critically ill patients.

However, several limitations should also be acknowledged. The retrospective single-center design may limit the generalizability of the findings. In addition, although propensity score matching was used to reduce confounding, residual bias related to unmeasured variables cannot be completely excluded. Finally, microbiological resistance mechanisms were not evaluated at the molecular level, and treatment regimens were not standardized, which may have influenced clinical outcomes.

## 5. Conclusions

Beyond the implementation of intensive antimicrobial treatment, a comprehensive, multifaceted strategy is necessary to enhance patient survival in cases of *S. maltophilia* bacteremia. Our findings indicate that initial SOFA scores and lactate levels are significant indicators of mortality, but their predictive power is significantly enhanced when integrated into a comprehensive clinical model. The analysis also identifies hemodynamic shock and underlying malignancy as independent, potent risk factors that significantly multiply the odds of mortality. Therefore, a multidisciplinary management approach that prioritizes prompt hemodynamic stabilization and SOFA-based clinical surveillance is crucial. Finally, by shifting the focus from a purely pathogen-oriented treatment to a holistic, patient-centered clinical management strategy, clinicians can better navigate the complexities of *S. maltophilia* infections and improve patient outcomes.

## Figures and Tables

**Table 1:** Comparative analysis of the categorical variables based on grouping.

	Study Group (*n* = 148)					Control Group (*n* = 148)							
	Mean	Median	Min	Max	IQR	Mean	Median	Min	Max	IQR	U	Z	*p*
Age	67.77	70.5	20	96	17	65.58	69	21	99	24	10,200.5	−1.021	0.307
APACHE II	13.98	12	2	42	11	14.94	15	2	40	12	10,097.5	−1.164	0.244
SOFA	7.7	8	0	22	4	7.27	6	1	25	3	9529.5	−1.953	0.051
CCS	4.39	4	0	13	4	3.74	4	0	13	2	9778	−1.614	0.106
Dialysis duration	27.59	73	0	150	59	56.55	62	0	130	81	100,812	−1.379	0.168
Catheter duration	5.41	10	0	28	12	14.16	11	5	44	7	4805	−8.484	<0.001
Culture negative days	12.86	12	0	35	8	2.59	9	3	31	10	2050.5	−12.246	<0.001

Abbreviations: APACHE II, Acute Physiology and Chronic Health Evaluation II; SOFA, Sequential Organ Failure Assessment; CCS, Charlson Comorbidity Score; IQR, interquartile range.

**Table 2:** Comparative analysis of the continuous variables based on grouping.

	Study Group (*n* = 148)					Control Group (*n* = 148)							
	Mean	Median	Min	Max	IQR	Mean	Median	Min	Max	IQR	U	Z	*p*
Albumin 1	2.83	2.74	1.8	4.73	0.77	2.74	2.5	1.6	6	1.08	9316.5	−2.222	0.126
Protein 1	5.64	5.51	3.33	9.9	1.52	5.69	5.6	3.9	7.7	1.1	10,306.5	−0.877	0.38
ALT 1	45.31	24	7	324	38.25	101.22	21.5	7	452	26	9960.5	−1.349	0.177
AST 1	53	33	9	531	31.25	55.45	54.94	1.34	162.1	71.35	9671.5	−1.739	0.082
CRP 1	19.4	16.2	2.58	76	13.7	20.54	19.3	0.04	62.1	19.09	10,786	−0.225	0.822
Phosphorus 1	39.58	36	2.67	98	27.3	6.29	6	2	25	4	475	−14.257	<0.001
Calcium 1	7.91	7.94	5.2	10	0.96	7.89	7.5	5.8	9.8	1.15	9555.5	−1.899	0.058
Creatinine 1	2.77	1.82	0.2	11	3.53	1.94	1.45	0.27	7.29	1.67	10,067	−1.202	0.229
Lymphocyte 1	1338.21	1160	112	8500	1081.25	1349.03	970	50	11,200	950	9974	−1.328	0.184
Neutrophil 1	6702.3	5405	680	32,510	6397.5	7625.74	6240	330	31,740	8237.5	10,220	−0.994	0.32
WBC 1	9231.93	8165	1070	38,450	6982.5	10,046.61	8890	500	38,360	8405	9943	−1.37	0.171
Procalcitonin 1	38.06	35.5	2.38	75	25.83	16.02	2.3	0.01	128	16.69	3974	−9.482	<0.001
ESR 1	55.66	53	4	122	38.75	54.27	43.5	3	136	48.75	9992.5	−1.303	0.192
Lactate 1	3.61	3.1	1.2	12.5	1.73	3.47	3	0.75	13	1.6	9967.5	−1.338	0.181
Albumin 7	2.89	2.88	1.66	4.56	0.88	2.87	2.9	1.9	4	0.7	10,743	−0.284	0.776
Protein 7	5.64	5.64	3.56	8.5	1.29	5.56	5.71	3.32	9.41	1.13	10,466	−0.66	0.509
ALT 7	44.63	23	7	1210	25.75	47.87	24	4	1757	27	10,130	−1.117	0.264
AST 7	83.98	31	9	4596	39.5	50.13	37	9	704	33	9487	−1.99	0.047
CRP 7	8.58	5.05	0.2	178.9	8.23	6.08	3.7	0.6	35	3.93	9445.5	−2.046	0.041
Phosphorus 7	46.04	36	11.8	363	23	30.03	28.1	1.7	100	39.8	7292	−4.971	<0.001
Calcium 7	7.98	7.97	1	10.9	1.1	8.07	8.15	6.2	9.6	1.4	10,445	−0.689	0.491
Creatinine 7	2.73	1.37	0	12.2	3.78	2.73	2.2	0.11	10.9	2.77	10,482	−0.638	0.523
Lymphocyte 7	1006.48	790	102	3400	1083.25	796.51	745	70	3300	390	10,339.5	−0.832	0.405
Neutrophil 7	6415.31	5560.25	820	24,290	4140.98	6605.07	6213	912	15,447	5571.75	10,187	−1.039	0.299
WBC 7	9387.64	8685	1380	25,710	5347.5	9436	8685	1380	25,710	5347	10,876	−0.103	0.918
Procalcitonin 7	6.47	2.15	0.04	75	6.01	4.8	0.33	0.03	75	0.47	6605	−5.904	<0.001
ESR 7	42.91	36	1	149	49.5	42.64	28	4.2	161	43.05	10,461	−0.667	0.505
Lactate 7	2.84	1.64	0.18	13.6	2.19	2.55	2.15	0.01	11	2.05	9337.5	−2.193	0.028

Abbreviations: IQR, interquartile range; ALT, alanine aminotransferase; AST, aspartate aminotransferase; CRP, C-reactive protein; WBC, white blood cell count; ESR, erythrocyte sedimentation rate. 1: Day 1 (baseline); 7: Day 7 (follow-up measurement).

**Table 3:** The Chi-square results of the categorical data.

		Study Cases	Control Cases	Total	*x^2^*	*p*	Cramer’s V
Presence of malignancy	No	127	141	268	7.731	0.005	0.162
	Yes	21	7	28			
Dialysis requirement	No	91	99	190	0.941	0.332	0.056
	Yes	57	49	106			
History of catheter infection	No	130	143	273	7.967	0.005	0.164
	Yes	18	5	23			
Culture result	No growth	0	113	113	296	Not applicable *	1
	*S. maltophilia*	148	0	148			
	*E. coli*	0	11	11			
	Enterobacter	0	1	1			
	Klebsiella	0	6	6			
	Pseudomonas	0	2	2			
	*S. aureus*	0	10	10			
	Other	0	5	5			
Source of the culture sample	None	0	113	113	190.766	<0.001	0.803
	Blood	68	22	90			
	Catheter	38	13	51			
	Blood and catheter	42	0	42			
Hypotension	No	16	12	28	0.631	0.427	0.046
	Yes	132	136	268			
Shock	yok	84	20	104	60.718	<0.001	0.453
	var	64	128	192			
Outcome	Alive	78	74	152	0.216	0.642	0.027
	Deceased	70	74	144			

* 8 cells (50.0%) have an expected count less than 5. The minimum expected count is 0.50.

**Table 4:** (**A**). Comparative analysis of continuous variables based on outcome. (**B**): Comparative analysis of the continuous variables based on outcome.

**(A)**						
		**Median**	**IQR**	**U**	**Z**	***p***
Age	Deceased	72	16	9240.0	−2.316	0.021
	Alive	68	21.8			
APACHE II	Deceased	12	12	10,310.0	−0.864	0.388
	Alive	12	11.8			
SOFA	Deceased	8	5	6560.5	−6.019	<0.001
	Alive	6	4			
CCS	Deceased	4	2	10,068.0	−1.205	0.228
	Alive	4	3			
Dialysis duration	Deceased	0	25.8	10,435.0	−0.806	0.420
	Alive	0	48			
Catheter duration	Deceased	10	14	10,785.5	−0.219	0.827
	Alive	10	14.8			
Culture-negative days	Deceased	8	14	10,262.5	−0.956	0.339
	Alive	6	12			
**(B)**						
		**Median**	**IQR**	**U**	**Z**	***p***
Albumin 1	Deceased	2.7	0.7	10,231.0	−0.969	0.333
	Alive	2.6	1.02			
Protein 1	Deceased	5.58	1.2	10,331.0	−0.833	0.405
	Alive	5.6	1.3			
ALT 1	Deceased	22	26	10,369.0	−0.783	0.434
	Alive	23	32.3			
AST 1	Deceased	41	61.3	10,390.5	−0.752	0.452
	Alive	38	56.7			
Phosphorus 1	Deceased	23.3	34.5	9504	−0.196	0.5
	Alive	13.5	30			
Calcium 1	Deceased	7.7	1.22	10,341.0	−0.820	0.412
	Alive	7.6	0.81			
Creatinine 1	Deceased	1.48	2.8	10,892.0	−0.071	0.944
	Alive	1.63	2.19			
Lactate 1	Deceased	3.3	2	8479.0	−3.350	0.001
	Alive	2.8	1.48			
Lymphocyte 1	Deceased	980	850	10,449.0	−0.673	0.501
	Alive	1110	1234			
Neutrophil 1	Deceased	6030	8423	10,498.5	−0.605	0.545
	Alive	5705	6153			
WBC 1	Deceased	8370	9070	10,743.5	−0.272	0.785
	Alive	8530	6583			
CRP 1	Deceased	17	15.9	10,817.5	−0.172	0.864
	Alive	16.6	15.6			
Procalcitonin 1	Deceased	26	49.3	9401.5	−2.097	0.036
	Alive	19	37.9			
ESR 1	Deceased	52	46	10,407.5	−0.729	0.466
	Alive	47	41.8			
Albumin 7	Deceased	2.84	0.73	9955.5	−1.343	0.179
	Alive	2.98	0.82			
Protein 7	Deceased	5.7	1.1	9920.0	−1.392	0.164
	Alive	5.71	1.26			
ALT 7	Deceased	24	25.8	10,623.5	−0.436	0.663
	Alive	22	28			
AST 7	Deceased	35	38	10,623.0	−0.436	0.663
	Alive	32	33.5			
Phosphorus 7	Deceased	33.1	31.4	10,821.5	−0.166	0.868
	Alive	34	22.1			
Calcium 7	Deceased	8.1	1.14	10,662.0	−0.383	0.702
	Alive	8.1	1.26			
Creatinine 7	Deceased	2.47	3.8	8685.5	−3.069	0.002
	Alive	1.36	2.62			
Lactate 7	Deceased	1.99	2.59	9389.0	−2.113	0.035
	Alive	1.78	2			
Lymphocyte 7	Deceased	782	494	10,648.0	−0.402	0.688
	Alive	740	585			
Neutrophil 7	Deceased	5543	5301	10,258.5	−0.931	0.352
	Alive	5995	4886			
WBC 7	Deceased	8628	6450	10,363.0	−0.789	0.430
	Alive	8469	5935			
CRP 7	Deceased	4.1	8.47	9809.5	−1.541	0.123
	Alive	4	5.46			
Procalcitonin 7	Deceased	0.935	5.58	10,477.5	−0.634	0.526
	Alive	0.475	3.81			
ESR 7	Deceased	32.6	45.1	9763.0	−1.605	0.109
	Alive	26.6	48.5			

Abbreviations: APACHE II, Acute Physiology and Chronic Health Evaluation II; SOFA, Sequential Organ Failure Assessment; CCS, Charlson Comorbidity Score; IQR, interquartile range; U, Mann–Whitney U test; Z, standardized test statistic; ALT, alanine aminotransferase; AST, aspartate aminotransferase; CRP, C-reactive protein; ESR, erythrocyte sedimentation rate; WBC, white blood cell count. 1: Day 1 (baseline); 7: Day 7 (follow-up measurement).

**Table 5:** Correlation table of significantly associated clinical parameters.

		Age	SOFA	Presence of Malignancy	Shock	Culture-Negative Days	Catheter Duration	Phosphorus 1	Phosphorus 7	Lactate 1	Lactate 7	CCS	Outcome
Age	r_s_	1.000											
	*p*												
SOFA	r_s_	0.154 **	1.000										
	*p*	0.008											
Presence of malignancy	r_s_	0.023	0.065	1.000									
	*p*	0.700	0.262										
Shock	r_s_	0.075	0.098	−0.004	1.000								
	*p*	0.195	0.092	0.946									
Culture-negative days	r_s_	0.073	0.129 *	0.117 *	−0.211 **	1.000							
	*p*	0.213	0.026	0.044	<0.001								
Catheter duration	r_s_	0.103	−0.045	−0.104	0.375 **	−0.315 **	1.000						
	*p*	0.077	0.439	0.075	<0.001	<0.001							
Phosphorus 1	r_s_	0.048	0.142 *	0.177 **	−0.335 **	0.554 **	−0.334 **	1.000					
	*p*	0.412	0.014	0.002	<0.001	<0.001	<0.001						
Phosphorus 7	r_s_	0.083	−0.018	0.057	0.015	0.283 **	0.013	0.317 **	1.000				
	*p*	0.156	0.762	0.328	0.794	<0.001	0.830	<0.001					
Lactate1	r_s_	0.131 *	0.166 **	0.161 **	0.035	0.017	−0.068	0.098	−0.069	1.000			
	*p*	0.024	0.004	0.006	0.552	0.766	0.245	0.094	0.238				
Lactate 7	r_s_	−0.014	0.132 *	0.068	0.205 **	−0.030	0.119 *	−0.027	−0.206 **	0.124 *	1.000		
	*p*	0.805	0.023	0.240	0.000	0.606	0.041	0.645	<0.001	0.034			
CCS	r_s_	0.101	0.134 *	0.343 **	−0.016	0.030	0.144 *	0.117 *	0.068	0.087	0.055	1.000	
	*p*	0.081	0.021	<0.001	0.778	0.610	0.013	0.044	0.244	0.137	0.348		
Outcome	r_s_	0.135 *	0.350 **	0.194 **	0.334 **	0.056	−0.013	0.114 *	0.010	0.195 **	0.123 *	0.070	1.000
	*p*	0.020	<0.001	0.001	<0.001	0.340	0.827	0.050	0.868	0.001	0.034	0.229	

** The correlation is significant at the 0.01 level (2-tailed). * The correlation is significant at the 0.05 level (2-tailed). 1: Day 1 (baseline); 7: Day 7 (follow-up measurement).

**Table 6:** Logistic regression results of correlated data.

	B	S.E.	Wald	df	Sig.	Exp(B)	95% C.I. for EXP(B)	
							Lower	Upper
Age	0.010	0.009	1.215	1	0.270	1.010	0.993	1.027
Shock (1)	1.794	0.333	28.944	1	<0.001	6.014	3.128	11.561
Presence of malignancy (1)	1.462	0.561	6.784	1	0.009	4.314	1.436	12.961
SOFA	0.244	0.051	23.112	1	<0.001	1.276	1.155	1.409
Procalcitonin 1	0.014	0.006	5.503	1	0.019	1.014	1.002	1.026
Phosphorus 1	0.013	0.007	3.212	1	0.073	1.013	0.999	1.028
Lactate1	0.130	0.075	2.963	1	0.085	1.139	0.982	1.320
Constant	−4.617	0.763	36.609	1	<0.001	0.010		

B, unstandardized regression coefficient; SE, standard error; OR, odds ratio; CI, confidence interval. The reference categories for categorical variables are the absence of the condition (malignancy = absent; shock = absent). Hosmer–Lemeshow goodness-of-fit: *p* = 0.874; Nagelkerke R^2^ = 0.373.

## Data Availability

The original contributions presented in this study are included in the article/Supplementary Material. Further inquiries can be directed to the corresponding author.

## Supplementary Materials

The following supporting information can be downloaded at: https://www.mdpi.com/article/10.3390/pathogens15040364/s1, Table S1: Descriptive presentation of the continuous variables; Table S2: Descriptive presentation of the categorical variables; Table S3: Comparative descriptive table of the demographic, history and clinical data.
