# Supplementary material for: Independent Predictors of 28-Day Mortality and the Critical Role of Source Control in Stenotrophomonas maltophilia Bacteremia in the ICU
Source: Pathogens. 2026 Mar 30;15(4):364. doi: 10.3390/pathogens15040364 (PMC13119374; doi:10.3390/pathogens15040364)
Supplement: Supplementary file 1 [file pathogens-15-00364-s001.zip › pathogens-4226480-supplementary.pdf]

Table S1. Descriptive presentation of the continuous variables

	Mean	Median	Minimum	Maximum	Percentiles 25th	75th
Age	66.68	70	20	99	60	78
APACHEII	14.46	12	2	42	8	20
SOFA	7.48	7	0	25	5	9
Dialysis_duration	19.02	0	0	150	0	20
Catheter_duration	9.78	10	0	44	0	14
Culture_negative_days	7.57	6	0	35	0	14
Albumin1	2.79	2.63	1.6	6	2.3	3.2
ALT1	73.27	22.5	7	4520	12	43
AST1	67.88	42	2	531	19	99.5
CRP1	17.3	15.9	0.03	76	8.93	22.75
Phosphorus1	22.93	11	2	98	6	36
Calcium1	7.86	7.5	2.58	69	7.3	7.99
Creatinine1	2.21	1.29	0	11	0.58	3.22
Lactate1	3.54	3	0.75	13	2.3	3.9
Lymphocyte1	2011.39	1040	4.57	26950	560	1785.31
Neutrophyls1	7114.41	5200	68	54600	2327.5	9037.5
Procalcitonin1	27.04	22	0.01	128	2.29	42
Sedimentation1	48.35	45	3	122	26	69
Protein1	5.76	5.7	3.33	9.9	5.11	6.4
Urea1	84.55	74	1.4	350.96	49	103
WBC1	12505.63	11230	500	38360	8020	16182.5
Albumin2	2.88	2.9	1.66	4.56	2.48	3.24
ALT2	62.24	32	6	2929	18	57
AST2	67.05	34	9	4596	23	59
CRP2	7.33	4	0.2	178.9	2	9.07
Phosphorus 7	38.04	33.4	1.7	363	22.1	46
Calcium 7	8.1	8.12	1	10.9	7.5	8.8
Creatinin 7	2.55	2	0	12.2	1.01	3.5
Lymphocytes 7	901.49	760	70	3400	492.5	1040
Neutrophyls 7	9001.57	7200	820	27100	4410	13575
Procalcitonin 7	5.63	0.53	0.03	75	0.24	4.48
Sedimentation 7	36.68	23	1	149	13	56
Protein 7	5.81	5.89	3.49	9.9	5.2	6.43
Urea 7	79.14	64.6	9	304	44.9	98.86
WBC 7	12066.19	10025	1380	67900	7165	16260
Lactate 7	2.69	1.85	0.01	13.6	1.16	3.37

Table S2. Descriptive presentation of the categorical variables

		n	%
Gender	Male	118	39.9
	Female	178	60.1
Presence of a malignancy	No	268	90.5
	Yes	28	9.5
Dialysis requirement	No	190	64.2
	Yes	106	35.8
History of catheter infection	No	190	64.2
	Yes	106	35.8
Hypotension	No	28	9.5
	Yes	268	90.5
Shock status	No	104	35.1
	Yes	192	64.9
Culture result	No growth	113	38.2
	S.maltophilia	148	50.0
	E.coli	11	3.7
	Enterobacter	1	0.3
	Klebsiella	6	2.0
	Pseudomanas	2	0.7
	Staph.Aerius	10	3.4
	Other	5	1.7
Source of culture sample	No	113	38.2
	Blood	90	30.4
	Catheter	51	17.2
	Blood and Catheter	42	14.2
Outcome	Deceased	144	48.6
	Alive	152	51.4

Table S3. Comparative descriptive table of the demographic, history and clinical data

Variable		Study		Control			
		n	%	n	%	*p*	Cramer's V
Gender	Female	89	60.14	89	60.14	1	0
	Male	59	39.86	59	39.86		
Presence of Malignancy	Yes	21	14.19	7	4.73	0.005	0.162
	No	127	85.81	141	95.27		
Dialysis requirement	Yes	57	38.51	49	33.11	0.396	
	No	91	61.49	99	66.89		
History of Catheter Infection	Yes	18	12.16	5	3.38	0.005	0.164
	No	130	87.84	143	96.62		
Culture result	S.maltophilia	148	100	0	0		
	E.coli	0		11	7.43		
	Enterobacter	0		1	0.68		
	Klebsiella	0		6	4.05		
	Pseudomanas	0		2	1.35		
	Staph.Aerius	0		10	6.76		
	Other	0		5	3.38		
Source of sample*	Blood	68	45.95	22	14.86	***0.002***	***0.266 (moderate)***
	Catheter	38	25.68	13	8.78		
	Blood and Catheter	42	28.38	0	0		
	No growth	0	0	113	76.35		
Hypotension	Yes	132	89.19	136	91.89	0.427	
	No	16	10.81	12	8.11		
Shock	Yes	64	43.24	128	86.49	***< 0.001***	***0.453 (relatively strong)***
	No	84	56.76	20	13.51		
History of the condition							
Heart failure	Yes	28	18.92	6	4.05	***< 0.001***	0.233 (moderate)
	No	120	81.08	142	95.95		
Renal Failure	Yes	58	39.19	30	20.27	***< 0.001***	0.207 (moderate)
	No	90	60.81	118	79.73		
Bedridden	Yes	0	0	15	10.14	***< 0.001***	***0.231 (moderate)***
	No	148	100	133	89.86		
Acute abdomen	Yes	0	0	0	0	**-**	**-**

	No	148	100	148	100		
Alzheimer	Yes	3	2.03	1	0.68	0.314	
	No	145	97.97	147	99.32		
Burns	Yes	0	0	3	2.03	0.082	
	No	148	100	145	97.97		
Cardiology	Yes	33	22.3	15	10.14	***0.005***	***0.165 (weak)***
	No	115	77.7	133	89.86		
Congenital development	Yes	0	0	1	0.68	0.316	
	No	148	100	147	99.32		
COPD	Yes	26	17.57	12	8.11	***0.015***	***0.141 (weak)***
	No	122	82.43	136	91.89		
Dementia	Yes	14	9.46	2	1.35	***0.002***	***0.179 (weak)***
	No	134	90.54	146	98.65		
Diabetes	Yes	41	27.7	31	20.95	0.176	
	No	107	72.3	117	79.05		
Gastrointestinal	Yes	0	0	1	0.68	0.3161	
	No	148	100	147	99.32		
Hypertension	Yes	39	26.35	54	36.49	0.06	
	No	109	73.65	94	63.51		
Oncology	Yes	23	15.54	8	5.41	***0.004***	***0.165 (weak)***
	No	125	84.46	140	94.59		
Neurology	Yes	0	0	1	0.68	0.156	
	No	148	100	147	99.32		
Respiratory	Yes	0	0	2	1.35	0.156	
	No	148	100	146	98.65		
SARSCov-2	Yes	0	0	11	7.43	***< 0.001***	***0.196 (weak)***
	No	148	100	137	92.57		
Serebrovascular Event	Yes	11	7.43	10	6.76	0.821	
	No	137	92.57	138	93.24		
Trauma	Yes	4	2.7	6	4.05	0.52	
	No	144	97.3	142	95.95		
Other	Yes	0	0	4	2.7	***0.044***	***0.117 (weak)***
	No	148	100	144	97.3		
